# Directed aromatic functionalization in natural-product synthesis: Fredericamycin A, nothapodytine B, and topopyrones B and D

**DOI:** 10.3762/bjoc.7.171

**Published:** 2011-10-28

**Authors:** Charles Dylan Turner, Marco A Ciufolini

**Affiliations:** 1 Department of Chemistry, University of British Columbia, 2036 Main Mall, Vancouver, BC V6T 1Z1, Canada

**Keywords:** alkaloids, directed aromatic functionalization, fredericamycin, heterocyclic compounds, lithiation, nothapodytine, organolithium compounds, topopyrone

## Abstract

This is a review of our efforts toward the synthesis of a group of natural products that display noteworthy biological activity: Fredericamycin A, nothapodytine B, and topopyrones B and D. In each case, directed aromatic functionalization methodology greatly facilitated the assembly of the key molecular subunits.

## Review

Our laboratory is primarily interested in the total synthesis of natural products, and does not conduct research on directed aromatic functionalization ("DAF") per se. On numerous occasions, however, DAF technology has been key to the success of specific synthetic endeavors in our group. Herein, we illustrate the application of such techniques to three representative problems that we have addressed over the years: The syntheses of fredericamycin A, nothapodytine B, and topopyrones B and D.

### Fredericamycin A

**Scheme 1:**
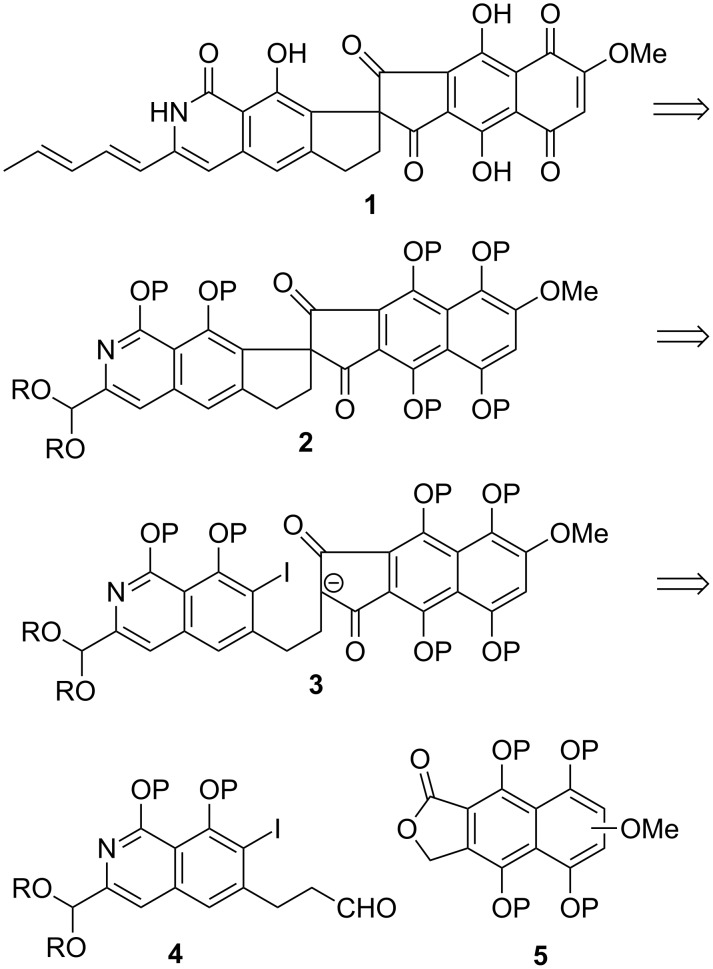
Structure and retrosynthetic analysis of fredericamycin A.

This initial portion of the present review recounts the first project that the senior author of this paper launched as an independent academic in 1984. A structurally novel natural product by the name of fredericamycin A (**1**, Scheme 1) had been discovered only two years prior and determined to be strongly cytotoxic [1–5]: A finding that spawned a flurry of activity in the synthetic arena. Indeed, seven total syntheses [6–16] and numerous approaches [17–30] have been described since. Fredericamycin seemed to be a superb vehicle to address an issue for which no solution existed at that time: The arylation of stabilized enolates. Indeed, the retrosynthetic analysis illustrated in Scheme 1 suggests that **1** could result from the cyclization of **3**, which in turn would ensue through the union of fragments **4** and **5**. These two subunits were prepared by DAF technology [31].

**Scheme 2:**
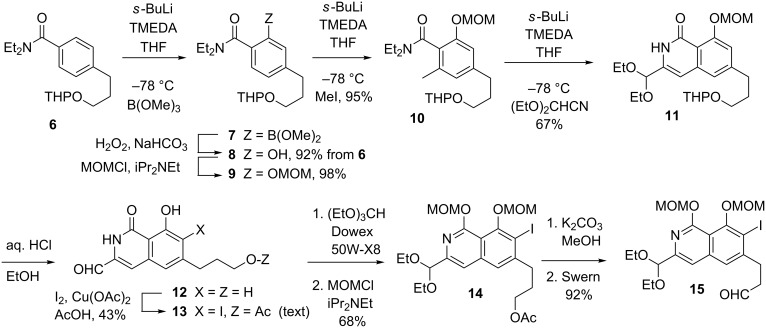
Assembly of the isoquinolone segment of fredericamycin.

The starting point for the assembly of an appropriate variant of **4** was diethylamide **6** (Scheme 2), which underwent smooth Beak–Snieckus-type *ortho*-deprotonation [32–35] with the *sec*-BuLi–TMEDA complex and consequent borylation in high yield. Oxidation of the ensuing **7** to phenol **8** and O-protection served as a prelude to a second *ortho*-deprotonation en route to methyl derivative **10**. A third round of *sec*-BuLi–TMEDA treatment induced highly selective deprotonation of the methyl group, and in keeping with the observations of Kelly [6–7], the intervening anion was intercepted by diethoxyacetonitrile [36] to furnish isoquinolone **11** directly. Relative to **4**, compound **11** lacks an iodo substituent, which was introduced by reaction of the free aldehyde **12** with I_2_ and Cu(OAc)_2_ in refluxing AcOH [37]. Only the unprotected **12** performed satisfactorily in this step, which, unfortunately, afforded an essentially 1:1 mixture of the desired phenolic *ortho*-iodide **13** and the corresponding *para*-isomer. These were readily separated after reprotection of the aldehyde and of the phenol, i.e., at the stage of **14**. Notice that in the course of the reaction the primary OH group in **12** was converted into an acetate ester (Fischer-type esterification). Compound **15**, a form of **4** suitable for the conduction of subsequent operations, was secured by deacetylation of **14** and Swern oxidation.

**Scheme 3:**
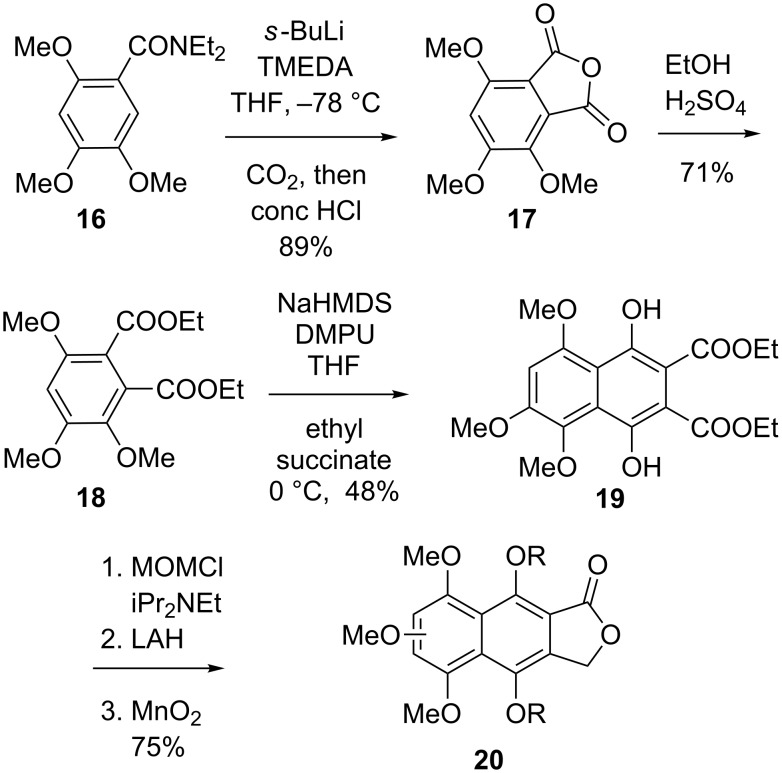
Synthesis of a naphthalide precursor to the quinoid moiety of fredericamycin.

Fragment **5** was best produced in the guise of compound **20**, the construction of which also relied upon DAF methodology. Thus, *ortho*-deprotonation of diethylamide **16** (Scheme 3) and carboxylation afforded anhydride **17** directly after workup with 12 N aqueous HCl. Fischer esterification and condensation of the resultant **18** with diethyl succinate, according to Kelly [6–7], afforded **19**, which was then elaborated to a 1:1 mixture of regioisomers of naphthalide **20**. It will be seen shortly that the formation of regioisomers at this stage is inconsequential.

**Scheme 4:**
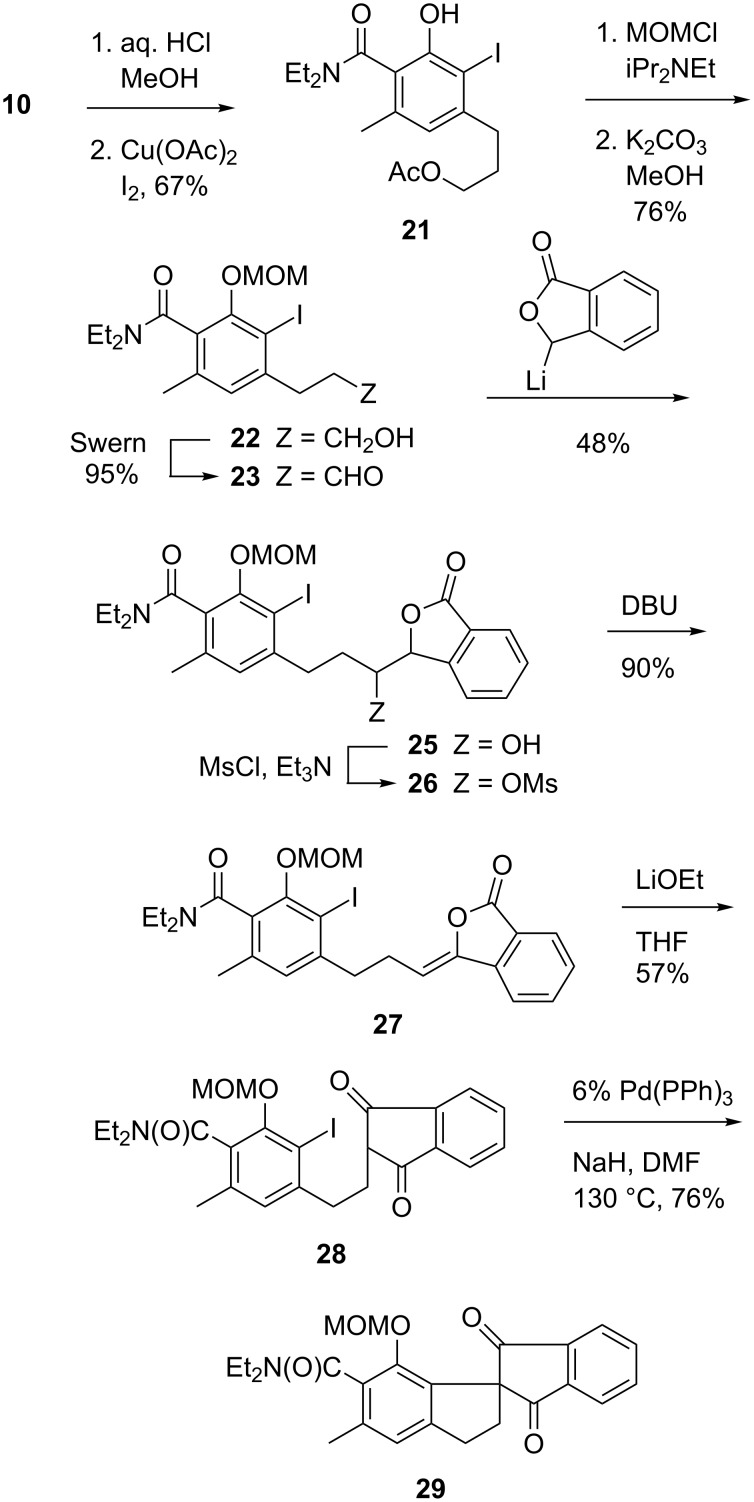
Palladium-mediated cyclization of a fredericamycin model system.

Parallel work had concentrated on defining a protocol for the merger of the two fragments, and especially for the conduct of the crucial, and the time highly novel, arylation step. The first issue, the more tractable of the two, was addressed by converting **10** into aldehyde **23** as a model of the more elaborate **15** (Scheme 4). Ironically, and contrary to the case of **12**, the iodination of this simpler system proceeded with superb ortho-selectivity to furnish **21** as the sole product. The aldehyde underwent tandem addition of the anion of simple phthalide (a model for **20**; prepared from the parent compound by deprotonation with LDA) and dehydration via mesylate **26** to provide **27**, which upon reaction with EtOLi (EtOH + BuLi) in THF rearranged to diketone **28**. Experiments involving **28**, as well as simpler model systems [38], revealed that the few techniques of enolate arylation then known were completely ineffective for the desired transformation. Remarkably, however, heating a DMF solution of the preformed sodium enolate of **28** (NaH) in the presence of Pd(PPh_3_)_4_ triggered cyclization to **29** in a very satisfactory 76% yield [39].

**Scheme 5:**
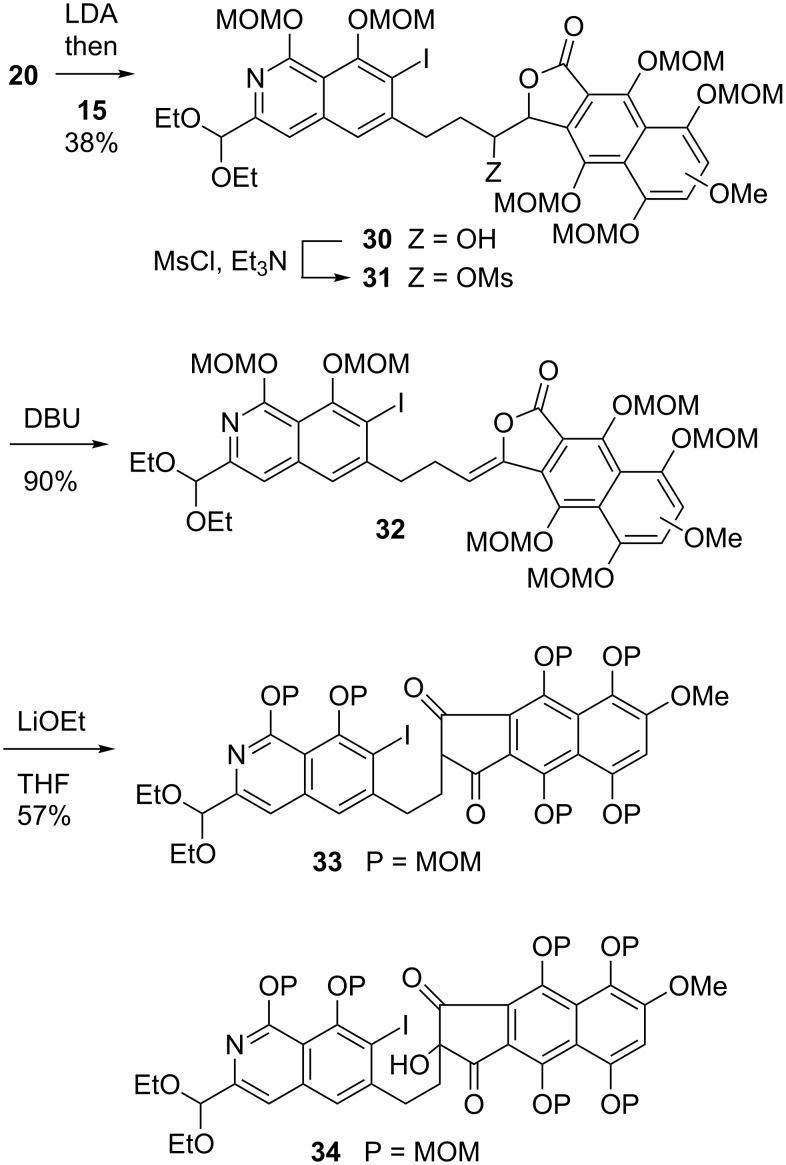
Synthesis of the precursor of fredericamycin and the facile air oxidation thereof.

On the basis of these results, the anion of naphthalide **20** was condensed with aldehyde **15** following the same protocol to yield compound **32** (Scheme 5). A distressing observation was made at this juncture: The base-promoted transposition of **32** to **33** was complicated by the unexpected propensity of the diketone to react with atmospheric oxygen to form the corresponding 2-hydroxy derivative **34**. This transformation was virtually instantaneous when the anion of **33** was exposed to the atmosphere, whereas **33** itself was completely hydroxylated in about 30 minutes at room temperature. Silica gel appeared to act as an effective catalyst for the process, further complicating purification, which ultimately had to be carried out in a glove box.

**Scheme 6:**
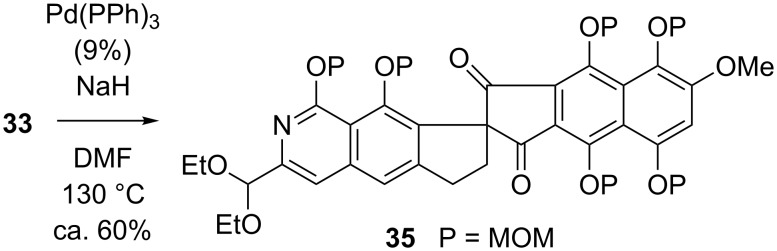
Formal synthesis of fredericamycin A.

Not without some difficulty, a batch of material consisting largely of **33** was subjected to the palladium-mediated cyclization reaction. This led to the formation of compound **35** in about 60% yield (based on ^1^H NMR; Scheme 6). A late intermediate in the Kelly synthesis of **1** [6–7] is structurally very similar to **35**. Consequently, the preparation of **35** corresponds to a formal synthesis of fredericamycin A [31]. In the years since, Pd-mediated arylation reactions of enolates have been extensively developed and improved, especially by Buchwald [40–43] and Hartwig [44–46].

### Nothapodytine

**Figure 1:**
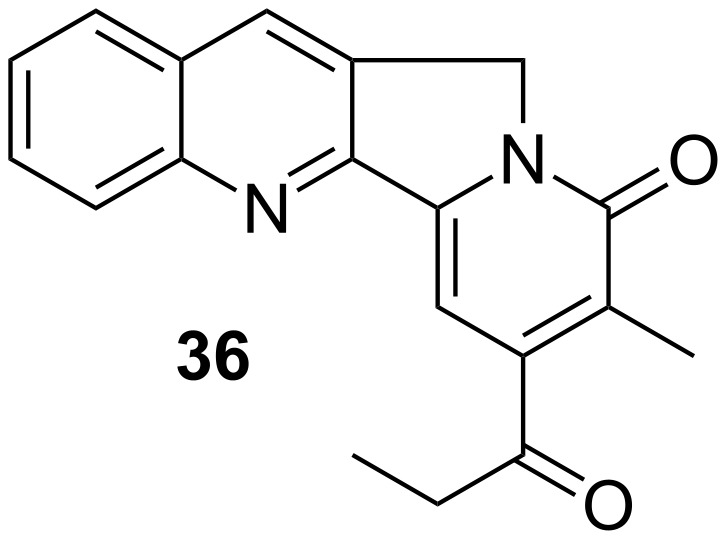
Structure of nothapodytine B.

Our group cultivates a long-standing interest in furan chemistry, especially in connection with a process that we describe as the “aza-Achmatowicz reaction” [47–52]: A transformation that has attracted the attention of a number of researchers worldwide [53–62]. In the course of such endeavors, we have often resorted to the facile C-2 deprotonation of furan as a means to generate appropriate derivatives. This is of course a special case of directed aromatic functionalization. A significant example of this chemistry was key to our synthesis of nothapodytine B, **36** (Figure 1) [63]. This substance is also known as mappicine ketone, in that it is an oxidized form of the alkaloid, mappicine [64], and indeed it can be converted into the latter by NaBH_4_ reduction of the ethyl ketone. A brief historical aside: The term “mappicine ketone” was apparently introduced by Kametani [65], who synthesized **36** in the course of investigations directed toward mappicine, and this more than 20 years before **36** was itself determined to be a natural product. In any event, nothapodytine exhibits interesting antiviral properties [66–67] and it has been the target of a number of total syntheses [68–74] and synthetic studies [75–79].

**Scheme 7:**
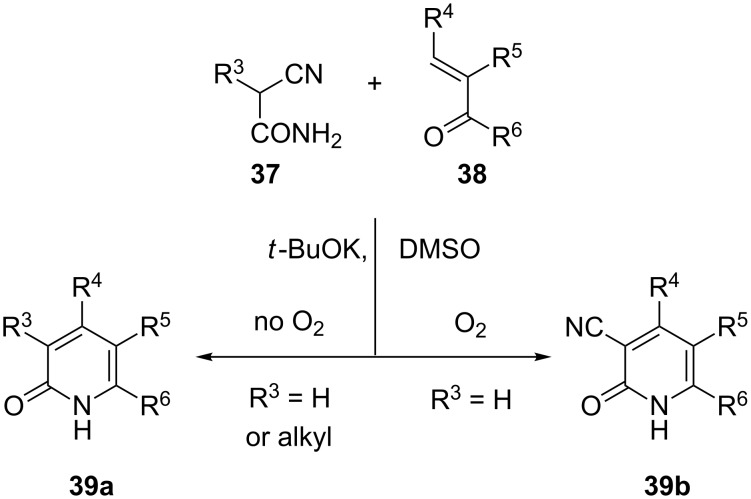
A useful pyridone synthesis.

The central problem we wished to address was the preparation of the 3-alkyl-2-pyridone moiety of **36** by an unusual [3 + 3] construction developed in our laboratory [80]. This chemistry (Scheme 7) promotes the condensation of a cyanoacetamide **37**, with an enone or enal **38**, in the presence of *t*-BuOK in DMSO. Under anoxic conditions, a series of events, culminating in the elimination of HCN from a presumed dianion intermediate, leads to the formation of pyridones **39a**, wherein R^3^ may be H, alkyl, or aryl. Conduct of the reaction with plain cyanoacetamide (cf. **37**, R^3^ = H) under an oxygen atmosphere produces cyanopyridones **39b** instead.

**Scheme 8:**
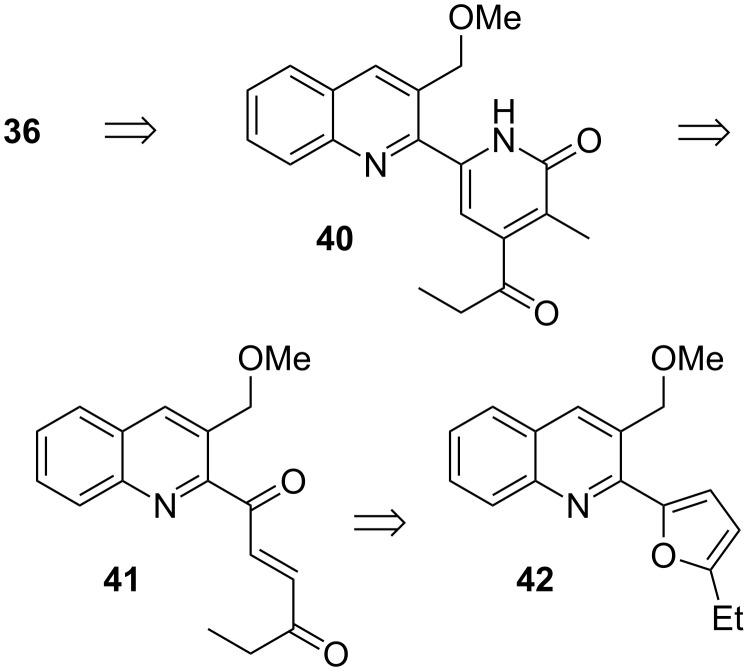
Retrosynthetic logic for nothapodytine B.

In keeping with a principle introduced during our work on camptothecin [81–83], the five-membered ring of **36** was imagined to result upon acid treatment of **40** (Scheme 8) [84], which in turn could be assembled by using the chemistry of Scheme 8 on substrate **41**, provided that the more electrophilic quinolyl ketone would direct an initial conjugate addition of the anion of a suitable cyanoacetamide to its own β-position. A particularly direct way to produce **41** seemed to be the oxidative cleavage of the furan ring in **42**, which thus became the first subgoal of our study. In that regard, we elected to prepare **42** by Suzuki coupling [85] between a furylboronic acid and a 2-chloroquinoline [86].

**Scheme 9:**
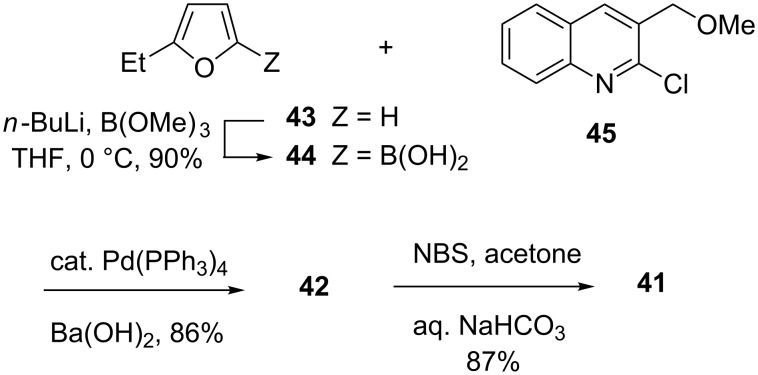
Preparation of a key nothapodytine fragment.

The (relative) acidity of the C-2 position of furan is such that *n-*BuLi without added TMEDA suffices to induce lithiation. Accordingly, treatment of commercial 2-ethylfuran with *n-*BuLi in THF at 0 °C and cannulation of the resulting mixture into a solution of trimethyl borate in THF afforded boronic acid **44** (Scheme 9) in 90% yield after the customary aqueous workup [87]. This material underwent smooth Suzuki coupling with the known **45** [81–83], and the action of aqueous NBS [88] upon the resultant **42** delivered the requisite **41** in 87% yield.

**Scheme 10:**
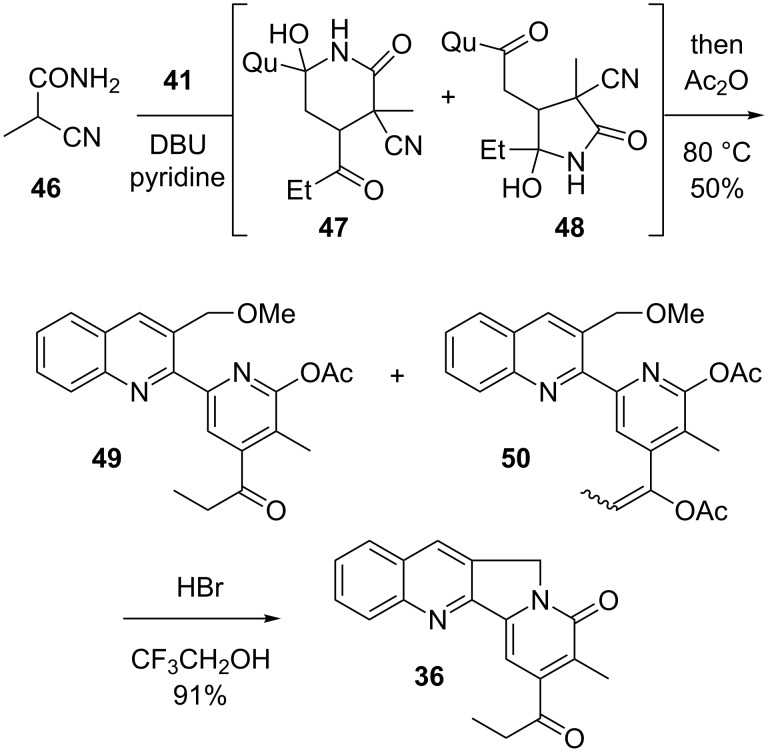
Total synthesis of nothapodytine B.

Happily, it transpired that the conjugate addition chemistry of **41** is indeed controlled exclusively by the quinolyl ketone. This enabled the conduct of our pyridone-forming sequence, which in the present case, however, had to be implemented in the slightly modified form seen in Scheme 10. Thus, Michael addition of 2-methyl cyanoacetamide converted **41** into a mixture of diastereomers of hemiamidals **47** and **48**. Attempts to force this mixture to advance to the desired pyridone under basic conditions yielded uniformly unsatisfactory results. This was due in part to undesired base-promoted reactions of the enolizable ketone segments, but also to the slow rate of interconversion of **47** and **48** (only the former can produce the desired pyridone). Experiment revealed that it was best to carry out the Michael step with DBU in pyridine, followed by addition of Ac_2_O and warming to 80 °C for an extended period of time. Under these conditions, a mixture of 2-acetoxypyridines **49** and **50** was obtained. Both compounds were transformed into **36** [89] upon exposure to HBr in CF_3_CH_2_OH (Boger conditions [68–69]).

### Topopyrones B and D

**Figure 2:**
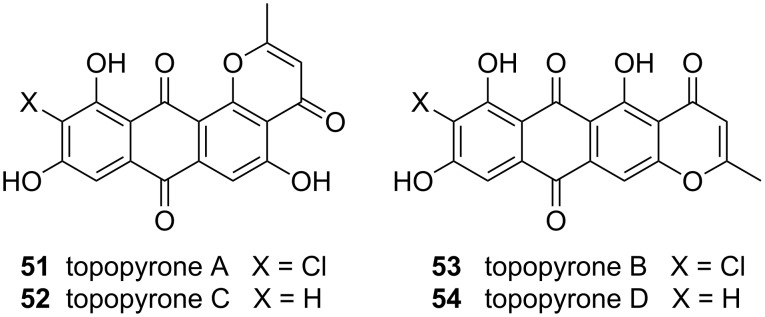
Structures of topopyrones.

Topopyrones A–D (Figure 2) were discovered during research aimed at identifying new topoisomerase inhibitors [90–91]. Initial evidence [90–91] suggested that these compounds may be selective inhibitors of topoisomerase-I (topo-I), a potentially significant finding. It should be noted that two topoisomerases are known, namely topo-I and topo-II. These nuclear enzymes relax superhelical tension in DNA during replication, transcription and repair. They operate by reversibly breaking one (topo-I) or both (topo-II) strands in double-stranded DNA and unwinding the severed strand(s), thereby relieving torsional energy. Inhibition of topoisomerases, which are overexpressed in cancerous cells, is fatal to the cell [92]. Subsequent studies revealed that topopyrones are in fact dual inhibitors of topo-I and topo-II [93]: A finding that diminished the biomedical relevance of the natural products. Regardless, the biological properties of topopyrones are sufficiently interesting that a number of groups embarked on a total synthesis [94–96].

Our own involvement in this area was motivated by an interest in topoisomerase-I inhibitors, which are important antineoplastic resources [97], the archetype of which is camptothecin [81–83,98–99]. The objective of the present effort was to obtain the target molecules by any means, as rapidly as possible, and in a fashion that might enable the production of analogs for structure–activity relationship studies. By contrast, our work on fredericamycin and nothapodytine had chiefly reflected a desire to illustrate applications of the new methodology to the synthesis of interesting natural products.

**Scheme 11:**
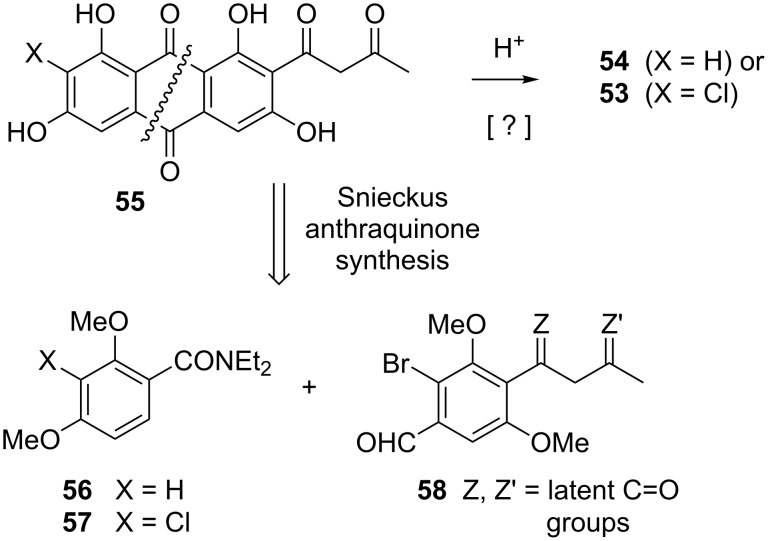
Retrosynthetic logic for the linear series of topopyrones.

The linearly fused topopyrones B and D are especially potent, and indeed, the activity of **53** against topo-I appears to be comparable to that of camptothecin [90–91,100–101]. Accordingly, our research centered on the linear series of compounds. A piece of information that was key to our retrosynthetic planning is that the action of alkali on **51** and **52** induces rearrangement to **53** and **54** [90–91], signifying that the linearly fused topopyrones are thermodynamically favored over their angular congeners. This implied that the cyclization of a precursor such as **55** under equilibrating conditions should selectively afford topopyrones B and D (Scheme 11). Drawing from the work of Snieckus [102–103], we further surmised that the assembly of **55** could be carried out in a single operation through the union of fragments **56** or **57** with aldehyde **58**: The carrier of a moiety that is common to all topopyrones.

**Scheme 12:**
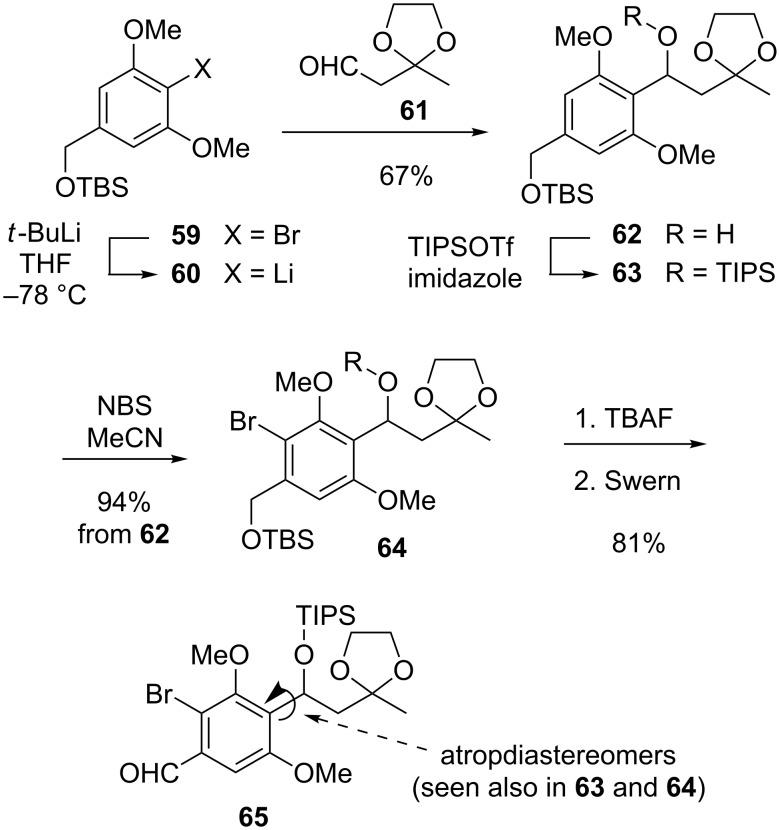
Construction of the molecular subunit common to all topopyrones.

A serviceable form of **58** proved to be compound **65**, the preparation of which is outlined in Scheme 12. A key step in this sequence was the addition of the organolithium species **60**, obtained through halogen–metal exchange of **59** with *t*-BuLi, to aldehyde **61** [104], leading to alcohol **62** in 67% yield. Straightforward manipulations of **62** then furnished the requisite **65**.

Interestingly, the ^1^H and ^13^C NMR spectra of all TIPS-protected compounds revealed that these substances exist as mixtures of atropdiastereomers. Furthermore, atropisomerism vanishes upon release of the TIPS unit (see below). An inspection of molecular models readily provides a qualitative illustration of this effect, in that the bulky TIPS group hampers rotation about the σ-bond connecting the benzylic carbon to the aryl segment (cf. arrows in **65**). An MM+ conformational study [105] of a simplified analog of **63** estimated an energy barrier for internal rotation equal to about 17 kcal/mol [106].

**Scheme 13:**
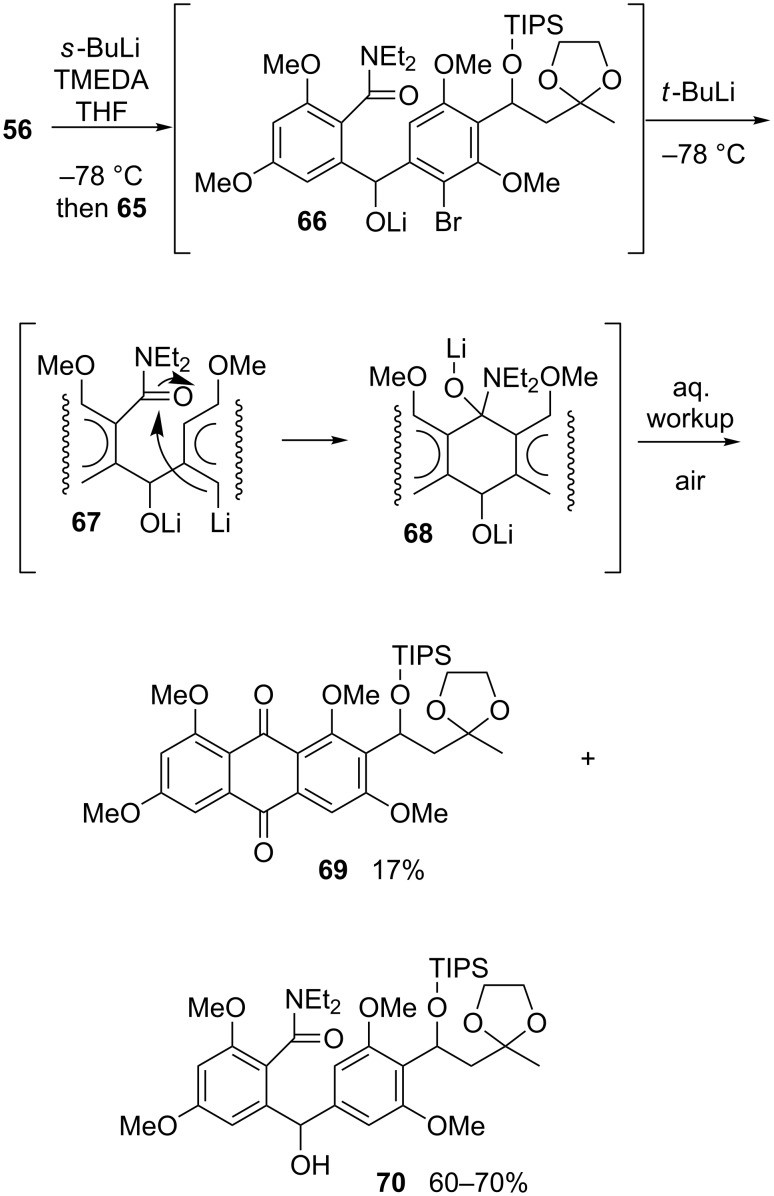
Difficulties encountered during the merger of the topopyrone D moieties.

As seen in Scheme 13, DAF technology came into the picture at the stage of the union of aldehyde **65** with amide **56**. Thus, lithiation of benzamide **56** with *sec*-BuLi/TMEDA (1.05 equiv, 3 h, −78 °C) and addition of the resulting organolithium agent to **65** was presumed to form the alkoxide **66**. In situ treatment of **66** with *t*-BuLi induced bromine–lithium exchange and cyclization of organolithium species **67** to a product that was believed to be **68**. Aqueous workup arguably converted **68** into a dihydroanthraquinone, which upon exposure to the atmosphere was rapidly oxidized to the desired anthraquinone **69**. The yield of this material was a modest 17% after purification, the remaining balance of the initial mass of **65** being the debrominated material **70** (60–70%). The NMR spectra of both products indicated that they existed as slowly interconverting atropdiastereomers, too. Numerous experiments aimed at improving the yield of **69** led to no fruitful outcome, revealing instead that the ratio of the two products remained essentially constant (ca. 1:3.5–4) regardless of the length of time (from 3 to 12 h) over which the reaction mixture was allowed to evolve following the addition of *t*-BuLi to **66**.

**Scheme 14:**
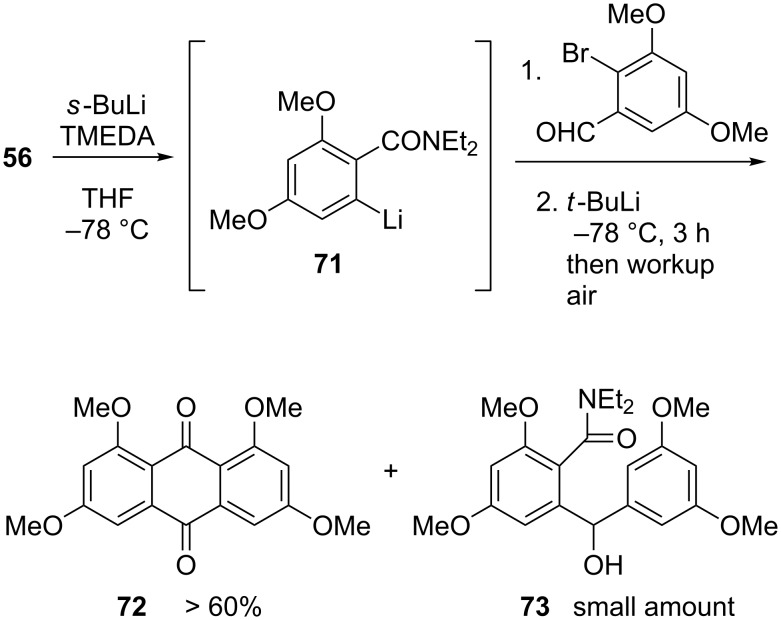
Efficient synthesis of a simplified anthraquinone.

As a control experiment, we examined the preparation of the simpler anthraquinone **72** by the same method (Scheme 14). This exercise established that **72** formed considerably more efficiently than **69** (over 60% isolated yield), and that it was accompanied by only small amounts of the debrominated byproduct **73**. We thus concluded that the low yield of **68** must have been the consequence of the consumption of a portion of aryllithium species **67** through parasitic proton-transfer steps, probably involving one of its benzylic positions as the proton donor. While the yield of **69** could not be improved, its preparation in the fashion just described is highly convergent. Furthermore, an overall yield around 20% for a one-pot sequence that involves three major steps (addition of lithiated **56** to **65**, halogen–metal exchange, cyclization of aryllithium species **67**) corresponds to an average of 55–60% yield per step. In such a light, the modest yield seemed quite acceptable, and indeed, with **69** in hand, the synthesis of topopyrone D was completed quickly.

**Scheme 15:**
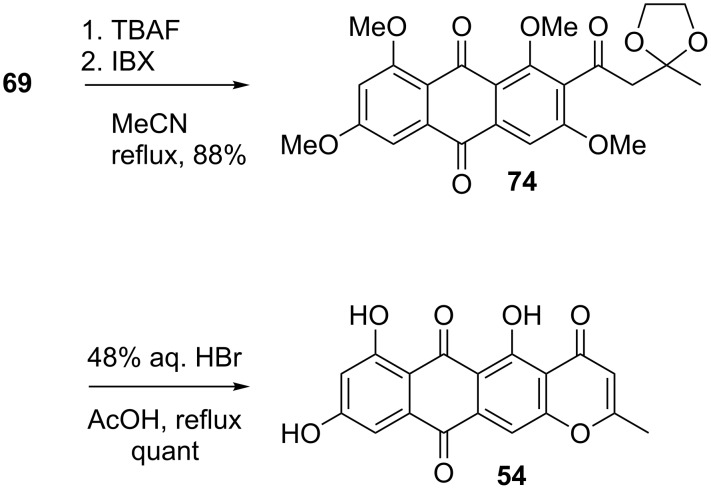
Total synthesis of topopyrone D.

As shown in Scheme 15, desilylation of **69** (TBAF) furnished an alcohol that, contrary to the parent **69** or other TIPS-protected synthetic intermediates, exhibited no atropisomerism (single compound by ^1^H and ^13^C NMR). Oxidation (IBX) and treatment of the emerging ketone **74** with 48% aqueous HBr in AcOH under reflux afforded synthetic **54** in quantitative yield. No evidence for the formation of angular topopyrones could be garnered, reinforcing the notion that the linear series is the thermodynamically favored one. Substance **54** was fully characterized as such, as well as the considerably more soluble triacetyl derivative, as detailed in the isolation paper [90–91].

An unexpected problem materialized when the foregoing sequence was transposed to the case of topopyrone B, the synthesis of which required the execution of the same operations starting with benzamide **57**. Surprisingly, this material resisted deprotonation under Snieckus conditions. For instance, treatment with five equivalents (as opposed to the customary 1.05 equivalents) of *sec*-BuLi–TMEDA complex for a prolonged period of time resulted in no incorporation of deuterium upon quenching with CD_3_OD. The reasons for this remain unknown to this date. Various chlorinated benzamides undergo *ortho-*deprotonation without difficulty [104], implying that the resistance of **57** cannot be attributed to the chloro substituent per se. Nor can the problem be ascribed to sequestration of the base through coordination/chelation [33] effects involving the chlorine atom. Such a hypothesis fails to account for the fact that **57** resisted deprotonation even in the presence of excess base. Moreover, 2,3,4-trimethoxybenzamide, a congener of **57** in which an OMe group replaces the Cl substituent, undergoes *ortho*-metallation without incident [107], even though the triad of adjacent OMe groups can surely sequester organo–Li species at least as effectively as the 2,4-dimethoxy-3-chloro arrangement present in **57**.

**Scheme 16:**
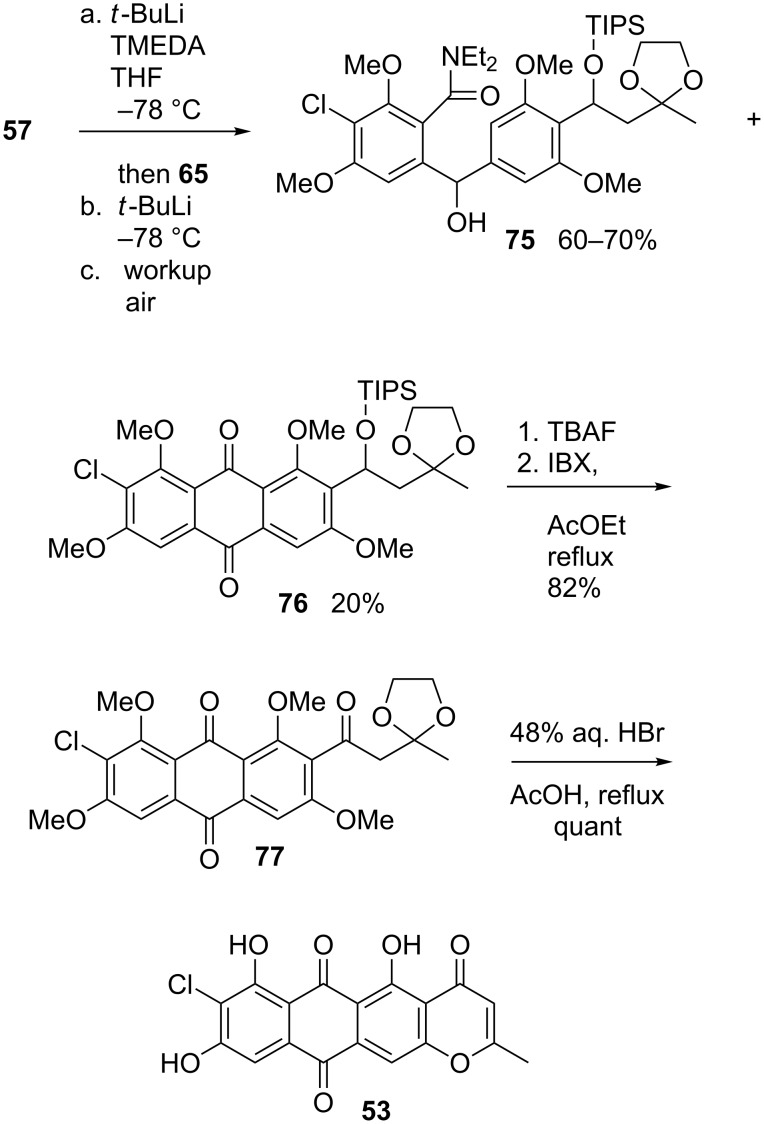
Total synthesis of topopyrone B.

In any event, *ortho*-deprotonation was ultimately achieved by the reaction of **57** with the more basic *t*-BuLi–TMEDA complex (1 equiv, 3 h, –78 °C; complete deuterium incorporation upon CD_3_OD quench). Accordingly, the organolithium species thus generated was processed as detailed earlier in Scheme 14, leading to a mixture of the desired anthraquinone **76**, obtained in 20% yield after chromatography, along with desbromo product **75**, again isolated in 60–70% yield (Scheme 16). The chemistry leading to **76** thus performed just as efficiently (inefficiently?) as before. The elaboration of **77** to the poorly soluble topopyrone B proceeded uneventfully, and in accordance with the isolation paper [90–91]; the ultimate **53** was most readily characterized as the trimethyl ether [108].

## Conclusion

The work summarized in this review exemplifies, hopefully in a convincing fashion, the value of directed aromatic functionalization in the preparation of complex aromatic systems. Indeed, it is difficult today to imagine an efficient route to natural products such as fredericamycin, nothapoditine, and topopyrones that eschews DAF technology altogether. More importantly, the chemistry described herein is a testimony to the far-sightedness of the pioneers in the field of directed aromatic functionalization. These giants have provided the chemical community with immensely powerful tools that have truly revolutionized the business of producing elaborate aromatic compounds, both in the academic laboratory and in the industrial plant, and that continue to produce countless benefits to society in the form of new medicines and new materials.
